# Dementia in the oldest old: Beyond Alzheimer disease

**DOI:** 10.1371/journal.pmed.1002263

**Published:** 2017-03-21

**Authors:** Aimee L. Pierce, Claudia H. Kawas

**Affiliations:** 1 Department of Neurology, University of California, Irvine, Irvine, California, United States of America; 2 Institute for Memory Impairments and Neurological Disorders, University of California, Irvine, Irvine, California, United States of America; 3 Department of Neurobiology and Behavior, University of California, Irvine, Irvine, California, United States of America

## Abstract

In a Perspective, Aimee Pierce and Claudia Kawas discuss risk factors and pathologies of dementia in the oldest-old.

## Introduction

The oldest old, people over the age of 90 years, are the fastest growing segment of the population worldwide. The medical problems facing the oldest old are unique in their scale and complexity and are only recently being studied in depth. Dementia is one of the most serious medical threats facing the oldest old, and although there is considerable variability in studies, several studies have noted that the incidence and prevalence of dementia continues to increase with advancing age, past the age of 90 [1,2,3,4,5]. Partially as a result of global disparities in lifespan, many of the current oldest old cohort studies are being conducted in developed countries in North America, Europe, and Australia [6], which may limit the wider applicability of the results across populations worldwide because of limited genetic and ethnic diversity in these countries. However, the oldest old continue to become more ethnically and racially diverse. For example, in the United States in 2012, 16.3% of the population 85 years and older was of minority background, which is projected to increase to 29.7% of minority background by 2050 [7]. Efforts are underway to study and harmonize data from more diverse oldest old cohorts [6].

There are several unique features to dementia in the oldest old. For example, risk factors (genetic and environmental) may differ in the oldest old, and the relative prevalence of dementia pathologies changes with advancing age. Moreover, the importance of mixed pathologies to the development of dementia increases greatly in the oldest old.

## Risk factors and prevention

Some of the risk factors for dementia in the oldest old are similar to those in younger adults, such as advancing age and low educational attainment [8]. However, some risk factors lose or change their association in the oldest old. For example, the APOE4 allele is less prevalent in the oldest old (likely because of a differential survival effect) and is not associated with incident dementia in the oldest old [9]. Similarly, midlife hypertension is a risk factor for dementia, but late-life hypertension appears to be protective against dementia in the oldest old [10]. Furthermore, the later the age of onset of hypertension, the lower the risk of dementia: Corrada et al. found in The 90+ Study that persons with onset of hypertension at age 90 or older had lower risk of dementia than those with onset of hypertension at ages 80–89 (total *n* = 559 initially without dementia, *n* = 224 who developed dementia during mean follow-up of 2.8 years) [11].

## Multiple pathologies

Recent research has identified that most cases of dementia in the oldest old are due to multiple pathologies, and these multiple pathologies may have deleterious combinatorial effects. Neltner et al. found 66% of individuals in this age group had two or more distinct brain pathologies identified at autopsy, and 27% had three or more pathologies (total *n* = 77, age range 98–107) [12]. Multiple pathologies greatly increase both the likelihood and the severity of dementia in this age group [13]. The combination of multiple pathologies challenges the investigation of links between pathology and cognition, diagnostic accuracy, and the development and testing of treatments for the oldest old. Understanding the relationships between pathology and cognition in the oldest old will require large cohorts (or more likely collaborations) with harmonized quantitative pathologic and cognitive measures.

## Non-Alzheimer pathologies

Some dementia-related pathologies are infrequent in younger populations but become more common in the oldest old. Examples include hippocampal sclerosis of aging (HS) and primary age-related tauopathy (PART). Hippocampal sclerosis of aging is defined by neuronal loss and gliosis in the hippocampal formation, not readily ascribable to another pathology such as neurofibrillary tangles or cerebral infarction, and usually (up to 80%) associated with TDP-43 immunoreactive inclusions [14]. HS has been seen in 5%–30% of the brains of oldest old and increases with age [14]. HS is highly associated with cognitive impairment. Although TDP-43 reactivity is also seen in frontotemporal lobar degeneration (FTLD), cases of FTLD-TDP-43 are exceedingly rare in the oldest old.

PART is characterized by subcortical and/or hippocampal neurofibrillary tangles without amyloid plaques, and there has been uncertainty as to whether it is a distinct pathologic entity or whether it will inevitably progress to Alzheimer disease (AD). PART was previously referred to as “tangle-only dementia”; however, it is now clear than in many cases cognition may be normal, or cognitive impairment may only be mild. Crary et al. found that 88 patients with definite PART on brain autopsy had final mean Mini-Mental State Examination (MMSE) (maximum = 30) scores ranging from 24–28, with higher Braak stages correlated with worse cognition [15].

Vascular pathology also appears to have increasing prevalence and relationship to dementia in the oldest old. For example, in The 90+ Study, 51% of participants had microinfarcts on brain autopsy, and the presence of three or more microinfarcts was strongly associated with dementia (total *n* = 213, dementia *n* = 110, odds ratio [OR] = 4.75) [16]. However, there is a great variety of vascular pathologies as well as measurement techniques, making comparison of the prevalence and association with dementia challenging between studies. There is also a link between HS and vascular pathology, particularly arteriolosclerosis [6]. In fact, one hypothesis is that HS may be caused by chronic age-related vascular pathology.

Another challenge of studying the underlying causes of dementia in the oldest old is the lack of biomarkers for many of these critical pathologies. Currently, it is not possible to identify several pathologies in a living person, such as hippocampal sclerosis, microinfarcts, Lewy bodies, and the anatomic distribution of tangles. Recent technological advances have resulted in several Food and Drug Administration (FDA)-approved positron emission tomography (PET) tracers for the detection of cerebral amyloid deposits, and PET tracers for tau are in human studies. In the near future, other PET tracers and MRI imaging techniques sensitive to other pathologies are likely to become available. There is also potential for the development of blood-based or other systemic biomarkers of general neurodegeneration or specific dementia pathologies (i.e., serum measures of amyloid, tau, or neurodegeneration; or detection of Lewy bodies in the submandibular gland or the enteric nervous system.)

It is also important to note that the development of AD pathology is not universal in the oldest old, as 25%–50% of the oldest old have “low” or “none” neuritic amyloid plaque density (Consortium to Establish a Registry for Alzheimer’s Disease [CERAD] system) [12]. It appears that the prevalences of AD and of Lewy body pathology increase with age up to the age of 90, but plateau or perhaps even decline in the oldest old [1]. Furthermore, the association between amyloid pathology and dementia is weaker in the oldest old compared to younger populations.

## Conclusion

Further study of the unique risk factors and pathologies of dementia in the oldest old is critical to successful prevention and public health care initiatives. Notably, none of the pathologies are universal in the oldest old, so there is the hope that individually they can be studied and the risk factors for each can be identified. Ultimately, it is likely that prevention and treatment for dementia in the oldest old will differ from approaches in younger populations. Combination therapeutic approaches may be necessary. Future clinical trials for dementia will need to focus on this specific, and ever-growing, population. As more members of the world’s population are afforded the opportunity to live into a very advanced age, we must find ways to prevent, delay, and cure dementia in this important age group.

## Abbreviations

AD: Alzheimer disease
FTLD: frontotemporal lobar degeneration
HS: hippocampal sclerosis
PART: primary age-related tauopathy
PET: positron emission tomography
